# The first smut fungus, *Thecaphoraanthemidis* sp. nov. (Glomosporiaceae), described from *Anthemis* (Asteraceae)

**DOI:** 10.3897/mycokeys.41.28454

**Published:** 2018-10-11

**Authors:** Julia Kruse, Volker Kummer, Roger G. Shivas, Marco Thines

**Affiliations:** 1 Centre for Crop Health, Institute for Agriculture and the Environment, University of Southern Queensland, Toowoomba 4350, Queensland, Australia; 2 University Potsdam, Institute for Biochemistry and Biology, Maulbeerallee 1, D-14469 Potsdam, Germany; 3 Goethe University Frankfurt am Main, Department of Biosciences, Institute of Ecology, Evolution and Diversity, Max-von-Laue-Str. 9, D-60438 Frankfurt am Main, Germany; 4 Biodiversity and Climate Research Centre (BiK-F), Senckenberg Gesellschaft für Naturforschung, Senckenberganlage 25, D-60325 Frankfurt am Main, Germany; 5 Integrative Fungal Research Cluster (IPF), Georg-Voigt-Str. 14-16, D-60325 Frankfurt am Main, Germany

**Keywords:** Glomosporiaceae, host specificity, internal transcribed spacer, molecular phylogenetics, smut fungi

## Abstract

There are 63 known species of *Thecaphora* (Glomosporiaceae, Ustilaginomycotina), a third of which occur on Asteraceae. These smut fungi produce yellowish-brown to reddish-brown masses of spore balls in specific, mostly regenerative, plant organs. A species of *Thecaphora* was collected in the flower heads of *Anthemischia* (Anthemideae, Asteraceae) on Rhodes Island, Greece, in 2015 and 2017, which represents the first smut record of a smut fungus on a host plant species in this tribe. Based on its distinctive morphology, host species and genetic divergence, this species is described as *Thecaphoraanthemidis***sp. nov.** Molecular barcodes of the ITS region are provided for this and several other species of *Thecaphora*. A phylogenetic and morphological comparison to closely related species showed that *Th.anthemidis* differed from other species of *Thecaphora*. *Thecaphoraanthemidis* produced loose spore balls in the flower heads and peduncles of *Anthemischia* unlike other flower-infecting species.

## Introduction

*Thecaphora* species belong to the Glomosporiaceae (Urocystidales, Ustilaginomycotina). The type species is *Th.seminis-convolvuli* described from *Convolvulusarvensis* (Convolvulaceae) collected in France (Desmazièrs 1827). Until now, 63 species of *Thecaphora* have been recognised (Vánky 2012), infecting host plant species in 16 different eudicot families (Vánky and Lutz 2007, Roets et al. 2008, Vánky et al. 2008, Vánky 2012). Species of *Thecaphora* produce sori in flowers, fruits, seeds, stems, leaves or roots, often in galls or pustules. The granular to powdery spore balls are yellowish-brown to reddish-brown, but never black. The majority of *Thecaphora* species produce loose or permanent spore balls without sterile cells. An exception to this is *Th.smallanthi*, which was reported to have large spore balls with outer spores and an internal layer of hyaline (sterile) cells (Piepenbring 2001). Three species have single spores (not united in spore balls), namely, *Th.thlaspeos*, *Th.oxalidis* (Vánky et al. 2008) and *Th.capensis* (Roets et al. 2008).

The Asteraceae is the largest family of eudicots with an estimated number of 30,000 species (Funk et al. 2009). The Asteraceae is divided into 13 subfamilies, including four (Asteroideae, Cichorioideae, Carduoideae and Mutisioideae) that contain about 99% of all taxa. *Anthemis* is a large genus in the tribe Anthemideae (subfamily Asteroideae), along with *Cota*, *Gonospermum* (including *Lugoa*), *Nananthea*, *Tanacetum* and *Tripleurospermum* (Bremer and Humphries 1993, Oberprieler et al. 2009, Presti et al. 2010). Species of *Anthemis* are distributed in western Eurasia, including the Mediterranean region, northern Africa and a small part of eastern Africa (Oberprieler 1998, 2001, Oberprieler et al. 2009, Presti et al. 2010). There are 62 species of *Anthemis* in Europe. *Anthemischia* belongs to the section Chiae and is a Mediterranean species common on Rhodes Island, Greece.

About 20 species of *Thecaphora* infect host plant species in six tribes of the Asteraceae. Taxa of the tribes Astereae and Heliantheae in the subfamily Asteroideae are often hosts of several *Thecaphora* species. Some less species-rich tribes, e.g. Coreopsideae, Millerieae, Polymnieae and Cynareae (subfamily Carduoideae) are also hosts of *Thecaphora* species. The species of *Thecaphora* on Asteraceae have not been studied by molecular phylogenetic methods, in contrast to species of *Thecaphora* on Caryophyllaceae (Vánky and Lutz 2007), Polygonaceae (Vasighzadeh et al. 2014) and Oxalidaceae (Roets et al. 2008, 2012).

Plants of *Anthemischia* with distorted flower heads containing mostly ligulate (ray) florets and swollen peduncles were collected near Tsambika, Rhodes Island, Greece, in 2015 and 2017. The swollen flower heads contained reddish-brown granular to powdery spore ball masses, typical of species of *Thecaphora*. The aim of this study was to identify the fungus and to determine its taxonomic assignment based on morphological and phylogenetic analyses of the internal transcribed spacer (ITS, barcoding locus) sequence data.

## Materials and methods

### Specimens

Herbarium specimens (23) of *Thecaphora* on a range of host plant species from across Europe and North America were examined (Tables 1, 2). The ITS sequences of specimens available on GenBank (19) and published in previous studies (Table 2) were included in the phylogenetic analysis. The nomenclature of the host plant species follows Euro+Med PlantBase (http://www.emplantbase.org/home.html) and the nomenclature of the fungi is according to Vánky (2012).

The morphology of the spore balls and spores of one specimen (GLM-F112531) of *Thecaphora* on *Anthemischia* was microscopically examined at 1000× in 80% lactic acid heated to the boiling point on a glass slide. Measurements of 30 spore balls and 100 spores were made with the Zeiss AxioVision software and micrographs were taken with an Olympus FE-120 camera on a Seben SBX-5 compound microscope (Seben GmbH, Berlin). The measurements are reported as maxima and minima in parentheses and the means are placed in italics.

### DNA extraction, amplification and sequencing

Genomic DNA was extracted from 23 herbarium specimens of *Thecaphora* (Table 1) using the methods reported by Kruse et al. (2017). The ITS nrDNA was amplified by PCR as reported in Kruse et al. (2018), using M-ITS1 (Stoll et al. 2003) as forward primer and either smITS-R1 or smITS-R2 (Kruse et al. 2017) as reverse primer. The ITS of host plants was amplified using primer pair ITS1P/ITS4 (Ridgway et al. 2003) with an annealing temperature of 53 °C. The resulting amplicons were sequenced at the Senckenberg Biodiversity and Climate Research Centre (BiK-F, Senckenberg) using the ITS4 primer (White et al. 1990). Sequences were deposited in GenBank (Table 2).

### Phylogenetic analysis

In total, 42 ITS sequences from 21 *Thecaphora* species were used in the phylogenetic analyses. Sequences were aligned with MAFFT v.7 (Katoh and Standley 2013) employing the G-INS-I algorithm and leading and trailing gaps were trimmed. The resulting alignment length was 534 bp. The methods of phylogenetic analysis were according to Kruse et al. (2018) using Minimum Evolution (ME), Maximum Likelihood (ML) and Bayesian Inference (BA). *Thecaphoraitalica* and allied species were selected as an outgroup, on the basis of the phylogeny presented by Vánky and Lutz (2007). Host plant species determination was verified by comparison with published sequences from Asteraceae deposited in GenBank (https://www.ncbi.nlm.nih.gov/genbank/) using BLASTN (Altschul et al. 1997).

**Table 1.** Collection records for specimens of *Thecaphora* examined in this study.

Species	Host	Country	Location	Date	Collector	Herbarium accession no.*
* Thecaphora affinis *	* Astragalus glycyphyllos *	Slovenia	Lower Styria, region Savinjska, N of Ljubno ob Savinjii, trail to Mt. Greben Smrekovec-Komen from Primož pri Ljubnem, wayside, 46°24'21"N, 14°49'54"E, 1150 m asl	14 July 2015	J. Kruse	GLM F112522
	* A. glycyphyllos *	Germany	Saxony-Anhalt, SW of Zschornewitz, forestry trail nearby SW-shore of „Gürke“ (Zschornewitzer Lake)	26 June 2007	H. Jage	GLM F094059
* Th. anthemidis *	* Anthemis chia *	Greece	Island Rhodes, 3.5 km NE Archangelos, Tsambika, way up to monastery, northeastslope, 36°14'03"N, 28°09'19"E, 90 m asl	26 April 2017	V. Kummer	GLM F112531
* Th. haumanii *	* Iresine diffusa *	Costa Rica	Prov. Guanacaste, 6 km NW de la barrada de la Laguna de Arenal	1 April 1992	R. Berndt, M. Piepenbring	M 0236177
* Th. leptideum *	* Chenopodium album *	France	Lotharingia, Forbach, Kreuzberg Mt.	Aug.-Oct. 1912/1913	A. Ludwig	M 0230099
* Th. molluginis *	* Mollugo cerviana *	Romania	Bratovesti, Oltenia	15 July 1963	K. Lug. Eliart	M 0236178
	* M. cerviana *	Romania	Oltenia, Timburesti	19 Sept. 1958	L. Pop	M 0236180
* Th. oxalidis *	* Oxalis stricta *	Austria	Upper Austria, Braunau at Inn, Hagenau Inncounty, Hagenauer Street, wayside, 48°16'24"N, 13°06'03"E, 340 m asl	18 Aug. 2014	J. Kruse	GLM F112523
	* O. stricta *	Germany	Bavaria, Upper Franconia, Fichtelmountains, Fichtelberg, Sandgrubenway, cemetery, 605 m asl	17 Sept. 2012	J. Kruse	GLM F112524
	* O. stricta *	Germany	Saxony-Anhalt, county Anhalt-Bitterfeld, Bitterfeld-Wolfen, Mühlstreet, allotment garden area „Kühler Grund“, 51°37'23"N, 12°20'08"E	13 July 2014	J. Kruse & H. Jage	GLM F112525
* Th. pustulata *	* Bidens pilosa *	Puerto Rico, USA	Mayagüez	13 Mar. 1920	H. H. Whetzel, E. W. Olive	CUP PR000458
* Th. seminis-convolvuli *	* Convolvulus arvensis *	Germany	Saxony, Middlesaxony, Freiberg, Halsbrücker Street, roadside, 50°55'31"N, 13°20'56"E, 400 m asl	11 Aug. 2017	J. Kruse	GLM F112527
	* C. arvensis *	Germany	Hesse, c. 8.5 km SE Eschwege, Weißenborn, Sandhöfe, path, 51°07'35"N, 10°07'25"E, 250 m asl	22 July 2017	J. Kruse	GLM F112528
	* C. arvensis *	Germany	Saxony-Anhalt, SSE Seeben, at Franzosenstein, wayside	26 Aug. 2002	H. Jage	GLM F065278
	* Calystegia sepium *	Germany	Mecklenburg-Western Pomerania, county Vorpommern-Rügen, 1,5 km NE of Barth, Glöwitz, rest area, 54°22'15"N, 12°45'38"E, 0 m asl	24 Aug. 2014	J. Kruse	GLM F112526
	* C. sepium *	Germany	North Rhine-Westphalia, county Steinfurt, Rheine, castle grounds Bentlage, between parking area and Gradierwerk, 52°17'49"N, 07°25'11"E, 35 m asl	14 July 2017	J. Kruse	GLM F112529
* Th. seminis-convolvuli *	* C. sepium *	Germany	Schleswig-Holstein, county Schleswig-Flensburg, Schaalby, W of Winningmay, parking area at „Reesholm“, wayside, 54°31'44"N, 09°37'53"E, 2 m asl	30 Aug. 2014	J. Kruse	GLM F112530
* Th. thlaspeos *	* Arabis ciliata *	Austria	Tyrol, district Kufstein, county Walchsee, Kaiserwinkel, track from hickinghut towards Niederkaseralm, over Hintere Abendpoit, eastslope Mt. Hochköpfl, 47°41'25"N, 12°19'37"E, 1300 m asl	21 July 2014	J. Kruse	GLM F112533
	* A. ciliata *	Germany	Bavaria, Chiemgauer Alps, county Rosenheim, Priener Hut, track 8,20, way up towards Kampenwand, alpine meadow, 47°42'29"N, 12°19'27"E, 1570 m asl	18 July 2014	J. Kruse	GLM F112536
	* A. ciliata *	Germany	Bavaria, Chiemgauer Alps, county Traunstein, Priener Hut, track 8,20 towards Priener Hut, alpine meadow, 47°42'07"N, 12°20'36"E, 1310 m asl	19 July 2014	J. Kruse	GLM F112537
	* A. hirsuta *	Germany	Hesse, Meißnerfoothills, Werra-Meißner-county, Großalmerode, S of Weißenbach, “Bühlchen”, calcareous grassland, 51°14'55"N, 09°51'08"E, 500 m asl	13 June 2015	J. Kruse	GLM F112532
	* A. hirsuta *	Germany	Bavaria, county Donau-Ries, Harburg, N of Ronheim, dry grassland, 435 m asl	20 June 2013	J. Kruse	GLM F112534
	* A. hirsuta *	Germany	Bavaria, Upper Bavaria, county Weilheim, N of Pähl, E at Hartschimmelhof, N „Goaslweide“, wayside, 720 m asl	20 July 2013	J. Kruse	GLM F112535

* Acronyms: GLM = Herbarium Senckenbergianum, Görlitz, Germany; CUP = Plant Pathology Herbarium, Cornell University, New York, USA; M = Botanische Staatssammlung, Munich, Germany.

**Table 2.** Specimens and GenBank sequences used for phylogenetic analyses. Sequences generated in this study are shown in bold.

***Thecaphora* species**	**Host**	**Herbarium accession no.** *^1^*	**ITS GenBank accession no.**	**Reference**
* Th. affinis *	* Astragalus glycyphyllos *	GLM F112522	**MH399748**	this paper
		GLM F094059	**MH399749**	this paper
* Th. alsinearum *	* Stellaria holostea *	HUV 10535	EF200032	Vánky and Lutz 2007
* Th. amaranthi *	* Amaranthus hybridus *	HUV 20727	EF200013	Vánky and Lutz 2007
* Th. anthemidis *	* Anthemis chia *	GLM F112531	**MH399758**	this paper
* Th. frezii *	* Arachis hypogaea *	Sa-EM1*	KP994420	Cazón et al. 2016
		Cba-GD2*	KP994419	Cazón et al. 2016
* Th. haumanii *	* Iresine diffusa *	M 0236177	**MH399764**	this paper
* Th. hennenea *	* Melampodium divaricatum *	HUV 14434	EF200014	Vánky and Lutz 2007
* Th. italica *	* Silene italica *	HUV 20345	EF200026	Vánky and Lutz 2007
		HUV 20344	EF200025	Vánky and Lutz 2007
* Th. leptideum *	* Chenopodium album *	M 0230099	**MH399756**	this paper
* Th. melandrii *	* Silene alba *	HUV 12677	EF200024	Vánky and Lutz 2007
* Th. molluginis *	* Mollugo cerviana *	M 0236178	**MH399762**	this paper
		M 0236180	**MH399763**	this paper
* Th. oxalidis *	* Oxalis stricta *	GLM F112524	**MH399759**	this paper
		GLM F112523	**MH399760**	this paper
		GLM F112525	**MH399761**	this paper
* Th. oxytropis *	* Oxytropis pilosa *	Kummer P 1146/3*	KF640685	Kummer et al. 2014
		Kummer P 1146/2*	KF640684	Kummer et al. 2014
* Th. pustulata *	* Bidens pilosa *	CUP PR000458	**MH399757**	this paper
* Th. saponariae *	* Saponaria officinalis *	TUB 012796	EF200022	Vánky and Lutz 2007
* Th. schwarzmaniana *	* Rheum ribes *	BASU 4242	JX006079	Vasighzadeh et al. 2014
		KRAM F-49788	KF297811	Vasighzadeh et al. 2014
* Th. seminis-convolvuli *	* Calystegia sepium *	GLM F112529	**MH399742**	this paper
		GLM F112526	**MH399743**	this paper
		GLM F112530	**MH399744**	this paper
	* Convolvulus arvensis *	GLM F112527	**MH399745**	this paper
		GLM F112528	**MH399746**	this paper
		GLM F065278	**MH399747**	this paper
* Th. solani *	* Solanum lycopersicum *	HUV 11180	EF200037	Vánky and Lutz 2007
*Th.* sp.	* Rheum palmatum *	S. Wang 1991*	KJ579177	Piątek et al. unpublished
		Y. Wang 2013*	KJ579176	Piątek et al. unpublished
		HUV 21117	KF297812	Vasighzadeh et al. 2014
* Th. spilanthis *	*Acmella* sp.	AFTOL 1913	DQ832243	Matheny et al. 2006
* Th. thlaspeos *	* Arabis hirsuta *	GLM F112532	**MH399752**	this paper
		TUB 015857	KJ579178	Vasighzadeh et al. 2014
		GLM F112534	**MH399750**	this paper
		GLM F112535	**MH399751**	this paper
	* Arabis ciliata *	GLM F112537	**MH399753**	this paper
		GLM F112533	**MH399754**	this paper
		GLM F112536	**MH399755**	this paper

^1^ Acronyms: AFTOL = Assembling the Fungal Tree Of Life, http://aftol.org; BASU: Herbarium of Bu-Ali Sina University, Iran; CUP = Plant Pathology Herbarium, Cornell University, New York, USA; GLM = Herbarium Senckenbergianum, Görlitz, Germany; HUV = Herbarium Ustilaginales Vánky, deposited in BRIP = Queensland Plant Pathology Herbarium, Brisbane, Australia; KRAM F = Mycological Collection of the W. Szafer Institute of Botany, Polish Academy of Sciences, Kraków, Poland; M = Botanische Staatssammlung, Munich, Germany; TUB = Herbarium Tubingense, Eberhard-Karls-Universität Tübingen, Germany; * not deposited in any public herbaria.

## Results

### Molecular phylogenetic reconstruction

**Figure 1.**
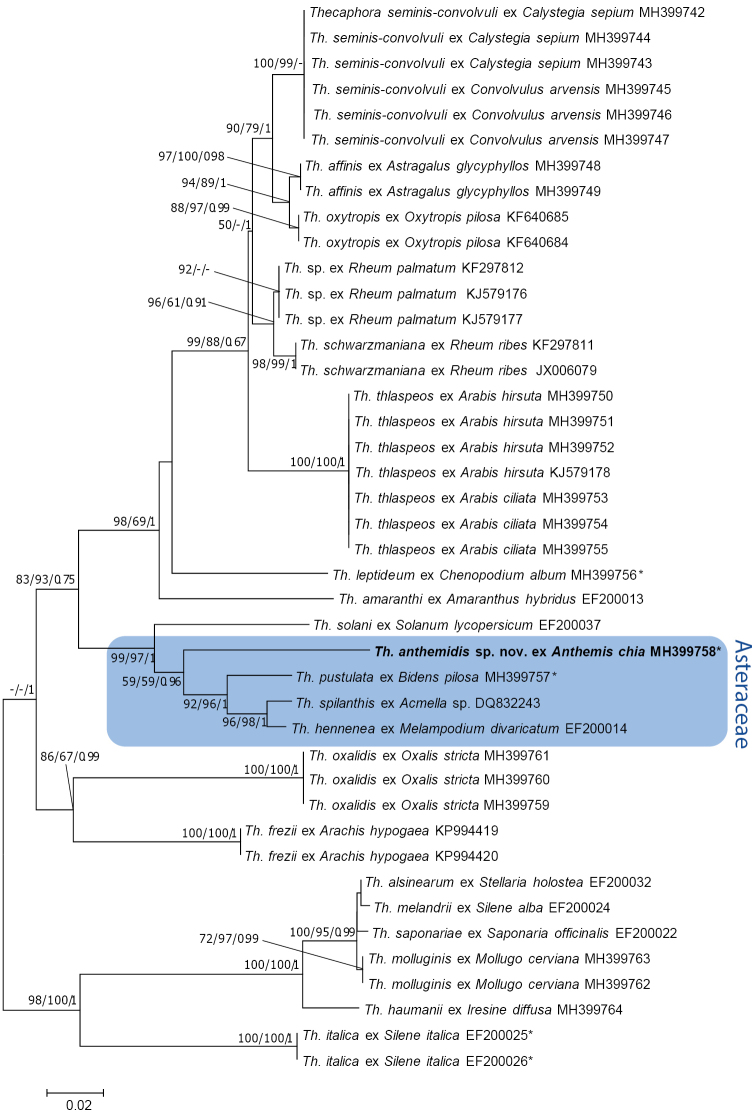
Phylogenetic tree of *Thecaphora* species based on ME analysis of the ITS locus. Numbers on branches denote support in ME, ML and BA, respectively. Values below 50% are denoted by ‘–‘. The bar indicates the number of substitutions per site. Ex-type sequences are highlighted with an asterisk.

The ML and BA trees yielded consistent topologies with the ME tree (Fig. 1). The *Thecaphora* sp. on *Anthemischia*, together with three Asteracious species (*Th.pustulata*, *Th.hennenea* and *Th.spilanthis*) and *Th.solani* from *Solanumlycopersicum* (Solanaceae), formed a sister clade to the species on other host plant families with strong to intermediate bootstrap support (83% in ME, 93% in ML). The *Thecaphora* sp. on *Anthemischia* was sister to the other Asteracious species with low bootstrap support (59% in ME, 59% in ML), but high Bayesian posterior probability (96%). The *Thecaphora* species on Fabaceae were polyphyletic, with *Th.frezii* on *Arachishypogaea* sister to *Th.oxalidis* on *Oxalisstricta* (Oxalidaceae). *Thecaphorafrezii* was distant to a monophyletic lineage on *Oxytropispilosa* and *Astragalusglycyphyllos*, which was sister to *Th.seminis-convolvuli*, the type of the genus. All specimens of *Th.seminis-convolvuli* collected on *Calystegiasepium* and *Convolvulusarvensis* (Convolvulaceae) had identical ITS sequences, as was the case with *Thecaphorathlaspeos* on *Arabishirsuta* and *A.ciliata* (Brassicaceae). Within the clade of mostly Caryophyllaceae-infecting species, two species of *Thecaphora* infected other families of the Caryophyllales, namely *Th.molluginis* on *Mollugocerviana* (Molluginaceae) and *Th.haumanii* on *Iresinediffusa* (Amaranthaceae).

## Taxonomy

### Thecaphora anthemidis

Taxon classificationFungiUrocystidalesGlomosporiaceae

J. Kruse, V. Kumm. & Thines sp. nov.

827067

Figure 2A–H

#### Type.

Greece, Rhodes Island, 3.5 km NE Archangelos, Tsambika, on path to monastery, northeast slope, 36°14'03"N, 28°09'19"E, 90 m a.s.l, on *Anthemischia*, 26 Apr. 2017, V. Kummer. Holotype GLM-F112531, isotype Herbarium V. Kummer P 1971/chia4; ITS sequence GenBank MH399758.

#### Etymology.

From the host plant genus *Anthemis*.

#### Description.

Sori in swollen and distorted flower heads and peduncles; spore ball mass initially white, later reddish-brown, granular to powdery; spore balls subglobose to ellipsoidal, rarely ovoid, mostly regular in shape, (31–) 36–*41*–47 (–52) × (28–) 31–*38*–44 (–50) µm, length/width ratio 0.9–*1.1*–1.2 (n=30), under light microscopy yellowish-brown to pale yellowish-brown, composed of 2–10 (–12) loosely united spores that separate easily; spores ellipsoidal, subglobose, ovoid or cuneiform, (18–) 20–*21*–23 (–25) × (14–) 17–*18*–20 (–23) µm, length/width ratio of 1.1–*1.2*–1.4 (n=100), with flattened contact surfaces and rounded exposed surfaces; wall at contact surface up to 0.5 µm thick, wall at free surface up to 3 µm thick, densely verrucose with warts 0.5–1 µm high, often confluent and sometimes irregular.

#### Host range.

*Anthemischia*.

#### Distribution.

Greece.

#### Notes.

*Thecaphoraanthemidis* has sori in the flower heads and the peduncles, which differentiates it from the following species that produce pustules, galls or swellings on the stems of Asteraceae: *Th.ambrosiae*, *Th.denticulata*, *Th.heliopsidis*, *Th.hennenea*, *Th.melampodii*, *Th.mexicana*, *Th.neomexicana*, *Th.piluliformis*, *Th.polymniae*, *Th.pulcherrima*, *Th.pustulata*, *Th.smallanthi* and *Th.spilanthis*. Four of the seven previously known species of *Thecaphora* that infect the flower heads of Asteraceae, namely *Th.arnicae*, *Th.burkartii*, *Th.californica* and *Th.cuneata* have firmly united spores that only separate after considerable pressure, which differentiate them from *Th.anthemidis* that has loose spore balls. Further, *Th.arnicae* (spore balls comprised of up to 25 spores), *Th.californica* (6–20 spores) and *Th.solidaginis* (8 to 50 or more spores) have larger spore balls with larger numbers of spores than *Th.anthemidis*. The spores of *Th.cuneata* are radially arranged within the spore balls and *Th.burkartii* has spores with an outer wall 5–9 µm thick, which is more than three times thicker than in *Th.anthemidis*. *Thecaphoralagenophorae* and *Th.trailii* are morphologically most similar to *Th.anthemidis*. *Thecaphoralagenophorae* is only known to infect *Solenogynegunnii* (tribe Astereae) in Australia (Vánky 2012). *Thecaphoratrailii* infects species of *Carduus*, *Cirsium* and *Saussurea* (Asteraceae, tribe Cynareae, Carduoideae) (Vánky 2012) and further differs from *Th.anthemidis* by having smaller spore balls (12–30 µm) and fewer spores (2–8) per spore ball.

**Figure 2.**
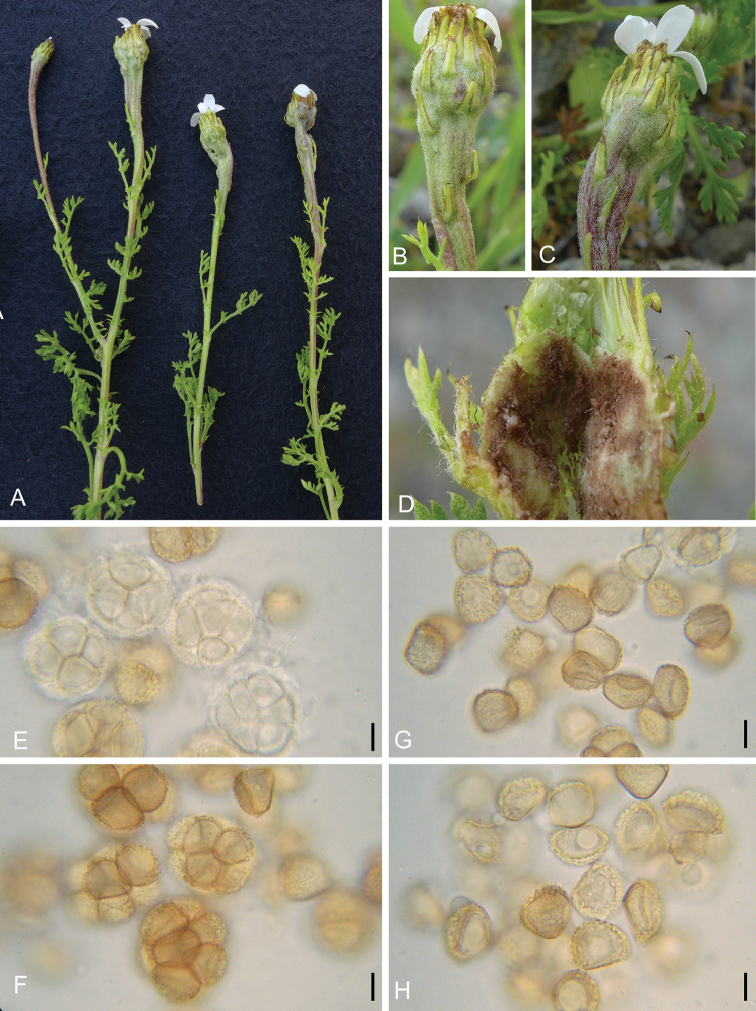
Sori, spore balls and spores of *Thecaphoraanthemidis* on *Anthemischia* (GLM-F112531) (**A–H**), **A** habit **B–C** swollen flower heads and peduncles **D** dissected flower head with reddish granular powdery spore ball mass **E** young spore balls **F** mature spore balls **G–H** single spores. Scale bars: 10 µm.

## Discussion

The present study is the first to identify a species of *Thecaphora* on a host plant species in the tribe Anthemideae (Asteraceae) (see Vánky 2012). *Thecaphoraanthemidis* was recovered in a monophyletic group of *Thecaphora* species on Asteraceae, sister to *Thecaphorasolani* on *Solanumlycopersicum* (Solanaceae). Our phylogenetic hypothesis, based on the ITS region, was similar to the analyses of the LSU locus of these taxa in Vánky and Lutz (2007) and Roets et al. (2008). In the latter study, *Thecaphorapolymniae*, which is known only from the type collection on *Polymniariparia* (Polymnieae, Asteroideae, Asteraceae) from South America (Vánky 2012), clustered within a clade of taxa that infect Fabaceae, Caryophyllaceae and Amaranthaceae (Roets et al. 2008). *Thecaphorapolymniae* has spores with a reticulate ornamentation and this may be evidence of a host jump from one of these plant families to Asteraceae. Host jumps have been reported before in the Ustilaginomycotina (e.g. Begerow et al. 2002, Piątek et al. 2017) and are thought to be a driver of plant pathogen diversification (Choi and Thines 2015).

Previously, only two ITS sequences of *Thecaphora* species infecting Asteraceae (*Th.spilanthis* and *Th.hennenea*) were available on GenBank, which together with the new sequences reported in this study, represents only 20% of all *Thecaphora* species known to occur on Asteraceae. In addition to the sequence of *Th.anthemidis*, we have provided barcode sequences of the ITS region for eight other taxa not previously available on GenBank (Table I). Future studies should address whether species of *Thecaphora* that infect the flower heads of Asteraceae form a monophyletic group.

## Supplementary Material

XML Treatment for Thecaphora anthemidis
